# New Elements of Operative Surgery

**Published:** 1846-07

**Authors:** 


ART. IV.—*New Elements of Operative Surgery.* By ALF. A. L. M. VELPEAU, Professor of Surgical Clinique of the Faculty of Medicine of Paris, Surgeon to the Hospital of La Charité, Member of the Royal Academy of Medicine, of the Institute, &c. Carefully revised, entirely remodelled, and augmented with *a Treatise on Minor Surgery.* Illustrated by over 300 engravings, incorporated with the text: accompanied with an *Atlas in quarto of twenty-two Plates,* representing the principal Operative Processes, Surgical Instruments, &c. First American, from the last Paris edition. Translated by P. S. TOWNSEND, M.D., late Physician to the Seamen's Retreat, Staten Island, New York. Augmented by the addition of *several hundred pages of entirely new matter,* comprising all the latest Improvements and Discoveries in Surgery, in America and Europe, up to the present time. Under the supervision of, and with Notes and Observations by VALENTINE MOTT, M.D., Professor of the Operations of Surgery with Surgical and Pathological Anatomy, in the University of New York; Foreign Associate of the Académie Royale de Médecine of Paris, of that of Berlin, Brussels, Athens. &c. In three volumes.—Vol. I, pp. 914: 1845. Vol. II, pp. 1092: 1846. New York: 8vo. J. & H. Langley.

Notwithstanding that this book has some rare claims on our attention, we had thought, owing to the late period at which the first volume reached us, to defer a notice of it until the whole should be issued. Having seen the second volume, however, and believing, as we do, that the translator and editor will kill the book off, will effectually damn it, in their intemperate zeal to give it currency, we have determined to come to the rescue and save it if we can. We are perfectly serious, of course.

We say it has rare claims to notice. It has passed through two or three editions in the original language. The author is one of the most remarkable men in the profession, and one of the most renowned. He is known to all tribes, kindreds, and tongues, by the many who have heard his voice in the lecture room, by the thousands who have been instructed by his pen. Of all his productions, numerous and excellent as they are, this is the one on which he himself seems to lay most stress, and the one, for aught we know, by which more especially he hopes to trasmit his name to posterity.

We repeat it has unusual claims to notice. It is sponsored by *MM.* Townsend and Mott, the latter of whom claims for himself (and "for his country," of course) the same position in American surgery that Velpeau holds in French. They pre-advised us long since of its preparation, lest it should take us unawares. They invited our attention to it in every possible way. The announcement has met the eye on the covers of medical journals; it has stared us in the face from publisher's catalogues; it has gone forth on the thousand wings of the daily press to every hamlet in the country. Nor did their exertions stop here. They were not satisfied with the *omne-ignotum-pro-magnifico* disposition of the common mind. They spoke, and still speak, of the work in the most magniloquent phrases; they have, in this respect, the most "damnable iteration" imaginable. If we adopt the opinion of the translator and his associate, repeated again and again in the most solemn and impressive manner, the world has never beheld such a "systematised condensation" of facts and experience, a surgical work so "thoroughly complete," so "judiciously classified," so "philosophically planned." A non-professional man, or even a professional novice, would suppose it to be the concentration, the essence, the summary, the epitome, or in the elegant language of the translator, the *abrégé*, of all that has been done, written, and said in and of surgery from the days of good-man Adam down to the present ten o'clock in the morning. He would look upon it as a veritable pocket Bodleian, a surgical Vatican in little. He would suppose no other book to be needed. He would conclude it to be as indispensable to the surgeon as eyes to a blind man, ears to a deaf one, or hands to an armless one. In short, he would be convinced that it not only pictured all the past and present phases of surgery, but adumbrated all the future.

It is evident that, in the opinion of the translator, genius can no further go—it is the *ultima Thule.* If it were an inspired work, he could scarce speak of it with more ardor. As there is one Iliad and one Homer, one Æneid and one Virgil, one Paradise Lost and one Milton, so there is one book on operative surgery and one Velpeau. And what Pope is to Homer, Dryden to Virgil, and Delille in France to Milton, so is Townsend to Velpeau.

But the translator has quite as exalted an opinion of surgery as of the book. It is spread over nearly the whole field of his mental retina. With him the part has become the whole, the less includes the greater. As Velpeau's work—that is to say, "this first American edition, as translated, and with notes, by Dr. Mott and myself"—supercedes every thing ever written, or ever to be written; so "is the great and controlling domain of Practical, Operative, and Pathological and Anatomical Surgery now monoplizing almost the entire healing art!" There is annexation for you with a vengeance. "The whole of Oregon, or none!" is the cry of the surgeons. Time was when there was "joint occupancy;" when physic and surgery had respective claims; when their domains as it were passed each other; when there was common ground. *Mais nous avons changé tout cela!* Surgery engulphs the whole, as easily as Aaron's rod swallowed those of the magicians. The surgeons claim the entire territory—"no compromise!"—54° 40′ !" To our translator "it is clear and indisputable" that Medicine is among the things that were—parodying Horace Smith—

"Physic is an idle waste of thought."

There are to be no more physicians—*non nominis umbra!* no obstetricians—no anatomists—no pathologists—it is *Troja fuit* with all these, except in a surgical point of view. Such of us as almost tremble at the name; who would as soon brave the hungry gaze of "a Hyrcan tiger" as the glitter of a polished catlin; who have been treating bilious fevers, agues, pleurisies, dysenteries, and Heaven knows what else, all our lives; who have been giving calomel and clysters, poking suppositories up the rectum and spreading blisters; and who never ventured further into surgery than doctoring claps and chancres—to find ourselves surgeons!—why it is a most rare circumstance! It is a joke that beats Moliere's *Médecin malgré lui* all hollow! It is most wonderful wonderful and yet again wonderful!

This putting a part for the whole, the less for the greater—this Synecdoche, as the Rhetoricians call it—is a favorite figure with our translator. He does not confine it simply to the book. Nor yet is he satisfied with taking surgery to be the entire healing art—the healing art "in its ensemble" as he would phrase it. In his enlarged comprehension he takes his "long-cherished, personal, and honored friend, preceptor and kinsman" (viz: *M.* Mott) to embody the whole of American surgery. Thus it is all in a concatenation accordingly—the consistency is beautiful—the correspondence admirable. The "Elements of Operative Surgery" is the *liber librorum*—

*Medicine is an idle waste of thought—*
AND MOTT IS EVERYTHING, AND EVERYTHING IS MOTT!

He is as much fascinated by him as a gaping boy, for the first time in an anatomical museum, would be fascinated by the rampant, six-foot figure of *papier maché* that graces the Medical Institute of this city. He cannot choose but gaze! and while gazing his honored friend grows under his sight as rapidly as Faust's poodle—he "grows long and broad!" "he fills the whole space!"—will the "*casus* make him laugh," when he sees "the kernel of the poodle?" Does the reader think that we exaggerate? Let him read! if he has a pair of lungs like a pearl-diver; but, if he is troubled with asthma, if in common parlance he is *phthisicky*, if he is old with the old man's wrinkles and anhelation, let him get some one to read for him, who will emphasize what we have placed in large capitals.

"But a still higher source of gratification I frankly confess, is that of the conviction, that in my attachment to a long-cherished, personal, and honored friend, preceptor and kinsman, and one whose eminent skill justly fills so large a space in Surgical history, (Dr. Mott,) I may have been, as I pledged myself I would be, (see Vol. I.) instrumental both for him, and for his country, in rescuing the details of all his great and master operations, and discoveries, and improvements in surgical processes and principles, from that indistinctness into which they were necessarily passing, by his neglect, (culpable it would be, but from his repugnance to self-laudation,) to gather them together as I now have done in these volumes, from the scattered Medical and Surgical periodical and fugitive works, in which they had been briefly registered, successively as they emanated from his great practical mind, or were created into existence by his INCOMPARABLE GENIUS and consummate skill.

"These master operations have been now, as will be seen in Dr. Mott's own special chapter on *Aneurisms*, also that of *Exsections*, &c., completely arranged and revised under his own inspection, in chronological order, so that hereafter there may be an orthodox work, to which reference may constantly be made for the authentic evidence of what he lays claim to as a Surgeon, and which, thus now, for the first time published to the world, in their *ensemble*, and under his sanction, together with his last, and latest opinions on these matters, present, in our view, we must confess, a precious treasure, for the student FOR ALL FUTURE TIME. It will, we feel assured, prove to be a great *organum* in Practical Surgery which the practitioner, as well as student, may never cease to peruse, and to re-peruse, as embracing some of the most important general *axioms* both in the minute manipulations and in the higher order of Surgical operations, and which will long continue to be received from so exalted a source, as among the surest and most practical land marks to guide those who aspire to eminence in this science, in their onward march to success and honor.

"I feel constrained also to add, that the accomplishment of this part of my labor, is one to which considerations of love of country add in my mind no slight degree of value. For young as our country is in history, and filled as its annals are with great names, both in the heroic periods of its settlement, and in its subsequent ages of military renown, national emancipation, and diffusion of the arts, there is in my humble judgment NO NAME THAT ADORNS THOSE ANNALS EITHER IN THE BATTLE FIELD, OR IN THE COUNCILS OF GOVERNMENT, OR IN ITS DI-

PLOMACY, that has added more sterling reputation, and abiding lustre to the intrinsic glory and future fame of America than that of *VALENTINE MOTT*(*!!!*), unaided and unguilded though that name may be by the insignia of office or of power."

How very solemn! We feel as if "the awful shadow of some unseen power" was over and round about us. We feel as *M.* Mott must have felt what time he journeyed in the East, when, after advertising the approach of the great American Surgeon to some minareted city, he passed through its streets thronged with the halt, the lame, and the blind, clamorous for his notice, and heard high above all the devout cry of some bearded and be-turbaned seller of fruit—"*In the name of the Prophet*, FIGS!"

Does the reader think this irony on the part of our translator? So would we, had we not two goodly volumes, with numerous expressions *passim*, all going to show his earnestness as if "he did'nt care who knew it." Does he think it most "admirable fooling?" Well, foolish it is beyond question, as bombastical as foolish, and as fulsome as the most pampered parasite, the most obsequious smell-feast could have made it. If the subject of such extravagant praises do not blush when he reads them, he is more or less than we take him to be. Will the reader have more? Let him read again, bearing in mind our caution.

"For one who in his duties as a christian and a man of science, has done so much personally in the active field of benevolence, and struck out such noble discoveries and new paths to enable us more effectually to alleviate the miseries of our fellow creatures, there will be a balm as we believe, that will rest upon his memory in after time, less dazzling perhaps, but certainly full as enduring, and far more endearing to future generations, than any that the most ardent homage of patriotism could feel inspired with, for the glare of the most brilliant civic or military exploits."

"Excellent! why this is the best fooling after all"—it would almost "draw three souls out of one weaver." Does any one ask now what this surgical Malvolio has done that he thus has so much greatness *thrust* upon him? or

> "Upon what *meat* doth this our Cæsar feed,
> That he is *grown* so great?"

Why he himself shall tell. He

"Has practised the ligature of the arteria innominata once, of the common iliac once, of the external iliac six times, of the internal iliac once, of the femoral artery forty-nine times, of the right subclavian within the scaleni muscles, once, of the right and left subclavian without the scaleni muscles, three [now four] times, of the primitive carotid, nineteen times, [now twenty,] and of the external carotid, twice. He has amputated [for osteo-sarcoma] the lower jaw nine times, two of which operations were at the temporo-maxillary articulation, and the upper jaw fourteen [sixteen] times. He has twice practised tracheotomy in the case of croup, [and a number of times for the removal of foreign bodies,] four [nine] times, the section of the sterno-mastoid muscle to relieve torticollis, once the extirpation of a thyroid gland that weighed five pounds two ounces, once [several times] the ligature of the thyroid arteries, with the view of causing atrophy of a goitre, once the exsection of the clavicle, several times the operation for empyema, once gastrotomy, one hundred and six times the operation for hydrocele with injection of sulphate of zinc, twice the exsection of the rectum, twice [several times] the extraction of loose bodies in the articulation of the knee-joint, many times the excision of false articulations, and once amputation at the hip-joint, and at the shoulder and the wrist, [four times operated successfully for the false articulations of the thigh by the seton, several times successfully on the tibia and other bones by the same mode, once successfully the restoration of an imperforate vagina to all its functions, in thirteen cases effected the perfect restoration of the functions of the lower jaw after years of permanent immobility, and once performed exsection of a portion of the nose and upper jaw for a large fibrous tumor]."

And all these cuttings—mere butcher's work at best—these mutilations, which, rightly considered, are so many *op-*

*probria* to the healing art; which, however necessary, are ever repulsive to the mind of him who is engaged in them, if that mind is properly constituted; these are to make this man

> ———"bestride the narrow world
> Like a Colossus, and we petty men
> Walk under his huge legs!"

these are to wrap up "his memory" after he dies, in some sort of phosphorescent "balm" (unknown, we apprehend, to the Pharaohs) until it

> ———"like a star,
> Beacons from the abode where the Eternal are,"

and flings a glory over his native country;—these, in short, are to make the LAND OF WASHINGTON known as the LAND OF MOTT!! Can folly go further? Would any man wish his worst enemy thus to stultify himself?

It is almost time for us to redeem the promise we made in the beginning, to save this book from its friends. But we cannot yet consent to leave our translator's preface—we should say prefaces, for, counting those in both volumes, there are three of them. They are in many things unique. They belong to a species altogether peculiar; they are indigenous to the region where we find them; and, for the sake of good taste, we trust they will never be naturalized elsewhere. We have not yet exhibited half of the beauties they contain. In variety of a certain kind, they cannot, whether taken individually or collectively, be equalled by anything we know of, not even Sheridan's speech on the Hastings' trial. We do not mean variety of style, for that is ever the same monotonous, involved, cumbrous, heavy-dragoon sort of thing; nor of eloquence, for we have quoted the most famous passages of that; nor of learning, there being scarcely that amount which is considered dangerous; nor of logic, for there is even less of that than of learning. We mean a variety in this wise—that one paragraph is apologetic, another explanatory; one laudatory, another condemnatory; one gratulatory, another monitory; one metaphysical, another metaphorical; one philosophical, another auto-biographical; whilst divers are self-abnegatory; and most are proud, vain, and fantastical. And these are "necessarily and naturally at times" all commingled and intermixed, like colors on a cloth where a painter wipes his brushes.

Shall we give a few more specimens? Here is one that might be termed analytico-laudatory. It shows not only nice discrimination on the part of the writer, but also great grammatical accuracy, upon which he plumes himself.

"The peculiar fitness which the author possesses for such an undertaking, is seen in the sound and well-disciplined mind with which he is gifted by nature—his ample education—his great professional ability and distinction as a practical operator and teacher of surgery—his practised pen as a man of extensive literary acquirements, together with his habits of untiring industry, patient research, and lastly, what is perhaps fully as important as all the rest, the clear judgment, the dignified impartiality, amenity of temper, and spirit of thorough philosophical investigation which he brings to every disputed or difficult question, or point, which is presented to his notice."

Why this is one of Shakspeare's men, anatomized in true Shakspearian style. What "a combination!" Are not the elements rarely mixed in him? Sits he not amongst us like a descended God? "Note him, good Charmian, 'tis the man; but note him!"

The first sentence of the next paragraph we do not know how to characterize, unless we call it philosophical. It strikes us that we have met with it before.

"The fruits of these rare intellectual and moral endowments, which are perhaps infinitely more desirable, certainly in general far more serviceable in society, than the irregular sallies and random efforts and effusions of mere isolated genius, are encountered upon every page of his work."

The careless reader may see nothing of particular moment in this little sentence. But to our thinking it contains the germ of a deep philosophy. Its half axiomatic structure gives point to a great truth it contains—a truth happily exemplified "upon every page" of these prefaces, which essentially consist of "the irregular sallies and random efforts and effusions of isolated genius."

The following sentence should be read as it stands in the book, in immediate connection with the one just quoted.

"As for example, whatever others may say to the contrary, the exact and full description which he gives in all the first portion of this volume in relation to MINOR SURGERY, or those common and indispensable operations, manipulations, dressings, &c., of a general though not difficult nature, which it is nevertheless important to be thoroughly conversant with as the rudiments of our art, and the only sure basis of all correct knowledge to be attained afterwards."

What we observe in this, besides the writer's usual grammatical accuracy, is his remarkable sagacity in nosing out the most important *fruit* of his author's rare intellectual endowments. From the prominence which he gives it, it is evident that he almost considers the treatise on Minor Surgery to be the crowning glory of "Velpeau's *great* elementary work."

"The professional reader also, whoever he may be, if imbued with the proper zeal and enthusiasm which should belong to him, will have reason to thank Professor Velpeau for the mass of valuable erudition which he has brought to bear by his unconquerable application and assiduity, upon the *true* history of every operative surgical process, principle, or discovery, which adorn or can illustrate the annals of the science, fortified as the whole also is by direct and specific references to every name, authority, or work, ancient or modern, upon which the proofs of his assertions and citations rest."

We have quoted this paragraph partly because it contains violations of syntax for which the writer would have been (and doubtless was) whipped when he was ten years old; and partly because the one we now give is dependent on it.

"We consider that this vein of rich, and much of it new historical contributions in surgery, and which so steadily courses or threads its way through the pages of the work, constitutes as it were the natural woof upon which the whole superstructure is woven, and thus forms to it a substratum of inappreciable value. Because it not only shows what has been done, and what has stood and still stands the test of time, and will doubtless continue to be approved of and recognised as of established utility; but it points out also as dangerous, if not forbidden paths to future explorers, those fruitless speculations and experiments that are to be avoided as not only a destructive waste of time to the student, but as exposing his mind to be seduced by visionary pursuits, and thereby into researches which will be liable to end in the repetition of similar abortive results."

This paragraph is only one of the many gems scattered through these prefaces, as diamonds are scattered in Golconda's mines. As a whole, it is tripartite in character, being metaphorical, scientific, and cautionary. Then the first sentence is likewise tripartite, being composed of three—nearly four—distinct metaphors, any one of which would suffice to make the fortune of an unknown writer. First, there is a "vein" filled with "new historical contributions"—a fluid which, though "quite a peculiar sort of juice" as Mephistophiles says of the blood, we agree with the writer in thinking decidedly "*rich.*" Then this *vein* "in a moment, in the twinkling of an eye," becomes a thread which we follow through the "dim cells and winding galleries" of these two volumes, as Theseus followed that of Ariadne through the labyrinth of Crete; until lo! it is multiplied into a "woof" so "natural" that we can almost hear the weaver's shuttle playing through it with alternating motion. The "woof," however, is but a transition stage—another metamorphosis and we stand upon a "sub-stratum," of a sort that would puzzle Lyell himself to describe.

Even without the charm of verse, which we are persuaded could easily have been given it, and which will be in the second edition, this is "perfect, past all parallel." It is the most remarkable instance on record of the ease with which things the most opposite and dissimilar can be fused into one homogeneous and sparkling whole. Genius is the only true amalgam, and the writer of this preface is its fortunate possessor. Did we believe in the transmigration of souls, we should insist that the soul of Ovid was now fluttering with restless wing within the mortal bodyof M. Mott's personal friend and kinsman, the translator of this "first American edition of Velpeau's great Elementary work on Operative Surgery." Insist upon this, did we say? we think better of it. We will maintain against the world in arms, that if Peter S. Townsend had lived in the first century after Christ, Publius Ovidius Naso, with all his harmonious and smoothly flowing numbers, would have been hissed and hooted from every bath and portico in Rome. This is our "sober second thought"—our deliberate opinion.

Let us, however, continue our examination. Enthusiasm sometimes, like murder, *will* out; and we found it impossible to wait until we had finished the examination of the whole paragraph, before we expressed our admiration of the writer's fancy and genius. In the second sentence, as near as we understand it, the vein re-appears. We left it a "substratum"—but, *quod fuit anté, reformet.* Soon, however, it experiences another astounding metamorphosis, being changed into something like a *finger-board*, and in this shape seems destined to be permanent—*armataque mansit imago.* As a finger-board it is quadripartite in its function—it points four ways at once, to "what has been done," to that which "still stands," to that which "will continue to be approved of," and lastly, to "speculations" and "experiments" which are converted into "paths," "primrose dalliance" in which is "to be avoided" as exposing to "visonary pursuits," whence would come "researches" ending in "similar abortive results." If the reader does not admire this, he must be as fastidious as a sick monkey. We *will* restrain any further expression of our admiration for fear of being thought crazy. Let, then, "expressive silence muse" the translator's praise.

Our next quotation is of the self-abnegatory and laudatory variety. The self-abnegation is positively excruciating, though it is by no means the best specimen we could find.

"We confess that it is with pride we present this work to the Profession; not so much, perhaps, from any pretensions we might make, in respect to the exactness and fidelity of the translation, or the composition and substance of the vast amount of new matter superadded, as from the conscious conviction we feel that there can be found nowhere, in any language, a surgical work so thoroughly complete, and so judiciously classified in (as has already been said in the Prefaces to the First Volume) the abundant and highly interesting and invaluable details which it comprehends, of surgical processes and discoveries, and surgical anatomy and relations, traced down with astonishing erudition and acute discrimination, by the learned author, Professor Velpeau, from the earliest epochs to the present day."

The construction of this sentence is altogether Ciceronian —it is musical in the extreme; but it is not this we wished particularly to notice. We also observe here the disposition of our translator to use the synechdoche. We have already seen how apt he is to put the part for the whole, the less for the greater. Here he puts the *thing containing* into the *thing contained.* He classifies a book in its details; whereas most authors attempt to classify details in a book.

But the point of most interest to us in this paragraph still remains. It is a new and very curious psychological fact discovered by our translator, viz: the existence of a "*conscious* conviction." Here is something that will astonish the phrenologists, and they are not easily surprised. It will dilate the eyes of a neurologist. It will even arch the eyebrows of a mesmerist. As yet this fact is a matter of personal experience with the writer; he "*feels* a conscious conviction;" but the evidence of so acute an observer will of course be taken as sufficient. The fact then exists. Is it a general one, or is it confined to this particular individual *homo*, making him "a marvel to the nations?" The answer is of immense importance. It would also be interesting to know in the present instance what the conviction is conscious of—whether, for example, of ignorance (as is not improbable), or crime, or inconsistency; nothing of this has been vouchsafed to us. We have tried our best to form some conception of this curious thing. The nearest approach we can make to it, is to imagine the feeling that a pregnant woman—"one who loves her lord"—may have, when near the end of her term. She has, it may be presumed, a *conviction* of her pregnancy; and if, as some contend, the yet unborn child is *conscious* (no matter of what) why of course the mother now and then *feels a conscious conviction.* Does the translator wish to create the impression that he is *pregnant?* His "estimable friend, M. Velpeau," some years ago found a man whom he pronounced "pregnant with his little brother." Is our translator that interesting individual? We pause for a reply.

We turn now to the auto-biographical portions of these unique prefaces. These we find scattered about like *disjecta membra*, and we have expended some labor in collecting them from the two volumes. This is a labor, however, which we have cheerfully performed, partly for the sake of the translator, partly for that of the profession of which we are both members, and partly for the sake of Dr. Mott's country, to which they will prove "a precious treasure," that "may be perused and re-perused." They may serve to infuse new energy into some flagging spirit; they may give heart to some "forlorn and shipwrecked brother." They are as few and as precious as the sybilline leaves; for our translator, like other men, is somewhat careless in this particular. He seems to forget that some of the interest which attaches to great men, attaches also to all those with whom they are connected; and that, however humble he may think himself to be (and really his humility is astonishing) and however humble he really may be, some of the interest which attaches to Mott, attaches likewise to himself. It is the quality of greatness to—

> "Spread undivided, operate unspent."

Men of such "incomparable genius" as Mott, are not only great in themselves, but they are the cause that greatness is in other men. Witness, for example, the case of Dr. John Murray Carnochan, who figures in these pages as a sort of metallic reflector of Dr. Mott's surgical light, partly because he, as well as Dr. Schmidt, claims to have been the first to divide the masseter muscle, but mainly because he was "one of Dr. Mott's pupils"—so it is carefully set down in the book, and in this particular he has a decided advantage over his competitor. Our translator, we say, has forgotten that, like some of the metaphors we have noticed, Dr. Mott is tripartite, or even quadripartite; that he is even more to him than Cerberus was to Mrs. Malaprop—"three gentlemen at once;" in short, that he is kinsman, preceptor, friend, and associate translator. Yet he has sometimes had this fourfold relation in view. Else we cannot account for the overwhelming impression made upon him by the man who did *not* first remove the lower jaw; nor for the 800-diameter-magnifying power of the lens through which he has gazed at the man who is the first ("in America") that amputated at the hip-joint, the first that sacrificed a black cock to Esculapius, and who is the first in the hearts of his countrymen. We may then "be doing a real and great good" in reminding him of it. We confess, moreover, that we are actuated by another motive—the fear, that, when the translator should recollect himself and not find these things noticed, he would "have it in a particular ballad else, with his own picture at the top of it."

But we will not detain the reader any longer from these interesting excerpts.

"I do not wish nor intend to dwell upon my own part in this performance, as I make no special pretensions to Surgery, however much I may admire the commanding solidity of its structure, over that of any other branch of Medicine, or however ready I may have been to avail myself of all the occasions which have presented themselves in my professional career, of indulging in a practical application of its principles, at least, to many of its minor operations and elementary processes.

"My professional labors have, it is true, been for the greater part of my life confined more to the practice of Medicine itself, strictly so called, and especially directed to the investigation of febrile diseases, as personally examined and investigated by me under all their varied aspects of climate and topography, and the influence of the latter on the human organization. I shall, however, never cease to reflect with pleasure, hard as the task has been, in the thought that in what concerns the great department of *Practical Surgery*, I shall have left some testimonials (lasting I trust) of my industry at least, and of my zeal to promote the advancement of art whose high utility all the world, *especially since its brilliant progress within* 30 *and even* 10 *years past*, now concede to be open to palpable and actual demonstration, and as indisputably entitled to be ranked as an exact science, and as far beyond the reach and assaults of empiricism as mathematics itself."

We have *italicised* a very interesting piece of information conveyed in the second pargraph. It will, we are persuaded, prove no less surprising than agreeable. What this brilliant progress of the world consists in—whether in accelerated motion around the sun or on its own axis, in morals or steam, in the arts of war or of peace—or in something entirely different—we are not informed. It is enough to know that the world has overtaken surgery, and conceded to it a proper rank. We somewhat congratulate ourselves that this forward movement dates from about the period of our nativity, and that its recent and marked acceleration is about co-eval with our manhood. We can scarcely persuade ourselves that these are mere coincidences.

There is another bit of information, dropped here by the translator, which, as being of less interest, we did not italicise. We mean the fact that reflection, which has heretofore been a hard task, will hereafter be easier. The mode of reflection "in the thought," is peculiar to him.

"I had also, in two different sojourns at Paris and Montpellier, in 1821–'22, and at Paris again in 1827–'28, had the happiness to become acquainted with, or to follow upon the clinical lessons of, some of the most illustrious of these men, as well as of their predecessors. It was natural that I should recur back with satisfaction of hours agreeably, if not profitably, spent under the instructions of such men as the Barons Larrey, Dubois the elder, Percy, and Dupuytren, Cuvier, Lacepede, Delpech, Richerand, Thenard, Gay-Lussac, Boyer, Roux, Lallemand, Civiale, Lisfranc, Cloquet, Velpeau, &c., &c."

Thus it is seen that, so far as preceptors are concerned, our translator has certainly been one of Heaven's favorites. Mott was the Alpha, Velpeau the Omega. Mott began the list, Velpeau ended it; so that, in reference to the equal greatness of the two men, we may say that Mott was the beginning of the end. But this is not all. The translator tells us in a note that at "the same sitting of the Academy" he absolutely *saw* La Place, Berthollet, Chaptal, Portal, Desgenettes, Legendre, and others! There is no telling what influence such a sight may have had on him. With such a galaxy of preceptors to teach the fledgling how to make his first essay, and with such incentives to sustained effort as were afforded by what he saw, it would be strange indeed if he had not reached his present exalted position before the world. Nature would deserve reprobation as a niggard of no ordinary character towards him. But his petals fortunately were not "nipt before they blew," nor "died they on the promise of the fruit." As if conscious of the interest that would be felt in him, he gives a list of part of his writings. It is with a feeling akin to indignation that we find this list in the degrading position of a foot-note printed in brevier.

We thus plant it, clothed in good small pica, at the top of the 67th page of the 6th volume of the Western Journal.

"I will refer here, to some of my principal writings on those subjects:

"1. My *Report* on the *Fever* which prevailed in *Bancker Street*, New-York, in the Summer and Autumn of 1820.

"2. Account of the *Yellow-Fever* of New-York, in 1822.

"3. Account of the Weather, Topography, and Diseases of the Bahama Islands, West Indies, 1823–'4–'5, published at New-York, 1826.

"4. Account of the Black Vomit, or Yellow Fever, at Havana, Island of Cuba, West Indies, 1830.

"5. Sundry Essays in various Medical Periodicals.

"6. My *Report* on the Yellow Fever, imported from the West Indies into Rondout, Ulster County, State of New-York, on the Hudson River, 100 miles North of New-York city, in the summer of 1843."

We regret that none of these productions have ever fallen under our notice. The editions were undoubtedly exhausted in the East. Still from the mere enumeration we have partly gathered and partly surmised two or three things. One is an auto-biographical item, viz: that our translator in the course of his varied life has worn palm-hats and smoked cigars in the West Indies, has eaten bananas and sucked oranges in their native clime. Another is a geographical fact, not noted in any work that we have seen, viz: that the position of the State of New York is on the Hudson River, 100 miles from New York city, in the summer of 1843. We commend this to Mitchell and Tanner.

This is positively all we know of the "birth, parentage, and education" of the translator of Velpeau's Operative Surgery. The account is lamentably meagre; it is unsatisfactory and provoking in the highest degree. We trust that he will yield to our earnest request to devote the preface of the third volume to his own glorification, as he has that of the first to the praise of Velpeau, and that of the second to the praise of Mott. He has apotheosised his two preceptors; he has placed them among the immortal; let him crown the work by "embalming" himself.

Let us pay our respects now to the preface of the associate translator. It is to him in the end that must be credited all the beauties and elegances we have thus far pointed out. He is the *final cause.* He advised and supervised the translation; he stands sponsor for Dr. Townsend's ability and faithfulness. All this the translator tells us repeatedly; it is a sort of feather in his cap. And all this the associate translator tells us in "his own special" preface; it is a sort of feather in his cap. This preface, whilst it preserves a striking resemblance to those we have noticed, has at the same-time something a little uncommon about it. This is as it should be. It would not do for one of such transcendent genius to write a common preface. If not exactly derogatory, it would not comport with true dignity. Moreover he must keep up the impression already made; he must continue to dazzle us. He must have an *epistolary* preface, for a reason very similar to that for which Bonaparte made the campaign in Egypt. This epistolary preface consists of two letters, one from *M.* Mott (thus we write him, and thus he likes to be written) to Velpeau, and one in reply from Velpeau to Mott—the whole eked out by a case of removal of the jaw, which M. M. thus takes occasion to relate *in limine.*

Of the first letter we may say what was said of the love-letter of a distinguished individual that "if not so good as we expected it is the best we ever heard." As there was a good surgeon spoiled when Dr. Townsend turned his attention to "febrile diseases, as personally investigated by himself;" so a great letter-writer was spoiled when his preceptor turned surgeon. We are convinced he might have achieved a great reputation in a difficult department of literature. He might have rivalled Walpole, Mad. Sevigne, Lady Montague, Pope, Byron, and many others, whose names we do not now remember. But in that case, the arteria innominata might never have been tied; certain "great and master operations" never been performed; divers "noble discoveries and improvements" never been "created into existence;" and sundry "new paths" never been "struck out." It is better therefore as it is. Though he might have witched the world as a letter-writer, it is better to have captivated his pupil's fancy and thrown a light of glory over his native country by cutting off legs and tying arteries. Meantime we have this epistle to show his versatility. It is an elegant specimen, and we should not be surprised if some day it figured in what is yclept the "Complete Letter-Writer," hawked about bar-rooms and steam-boat landings by dirty-faced and ragged little boys.

There is an old adage to the effect that "he who drives fat oxen, should himself be fat." It will be seen that Dr. T.'s preceptor fulfils all reasonable requirements in this particular. Many of the excellences pointed out in the pupil's prefaces, are also found in the preceptor's. Indeed there seems to be a very "sembable coherence" betwixt the two. There is the same happy tautology, the same inconsequence in many of their inferences, the same disregard of numbers and cases, and the same heaping of metaphor upon metaphor with most impossible conveyance. Moreover the sentences of each have the same sluggishness in getting towards a termination, dragging their slow length along like an Alexandrine or a wounded snake, as if the channels of thought through which they passed were convoluted like the intestines or the seminiferous ducts.

To show how fit a "sponsor" M. Mott makes, here are samples of the expressions he uses: "an inestimable *archive;*" "compendiums of a *limited* character;" "profited *of* opportunities;" "signify commendation *to* an undertaking;" "*mutually* acceptable to *both.*"

But here is a whole sentence—

"Because I felt that by associating my labors with those which have received the approbation of your judgment, and which your genius and untiring industry have wrought out in your estimable work, as an enduring monument to your fame, I might find an appropriate place and guarantee for indulging the ambition that I, like others, must naturally have of seeing legitimately transmitted, through an orthodox and approved organ, for the judgment of an impartial posterity, an authentic account at least (if only at best but an abrégé one) of my *stewardship* also in the great field of surgical science. This so far as concerns myself, (and independent of perhaps paramount considerations, to give wider diffusion upon this continent to your great and useful work,) I felt admonished by the time of life at which I have arrived, to be be due as well to myself and my own reputation, as to my conntry and profession."

We have already seen how the pupil applied the "flattering unction," and here we see how complacently the first preceptor takes it. He is charitable, too; he distributes it freely; and a little further on Velpeau repays it all liberally. In fact the harmony among the three is beautiful. They touch hands all round. First Townsend tickles (if we may use so familiar a phrase) Velpeau and Mott; then Mott tickles Townsend and Velpeau; then Velpeau tickles Mott and Townsend. We beg the Frenchman's pardon; but, for the nonce, we must take him in the company we find him.

It is evident, moreover, from this sentence, that the editor thinks himself all the translator says he is. He acknowledges no equal other than the author of these "Elements." He brooks no other rival near the throne. Velpeau is his *alter ego.* We can fancy him saying in the slightly changed language of rare Ben Jonson—

> ———"Great and high,
> The world knows only two, *Velpeau* and *I.*"

He will form a surgical *duumvirate.* He will share the world with the great Frenchman, but with nothing meaner. He will give him the Eastern continent, whilst he himself—the great American Surgeon—takes the Western. Or to express it in the language of old Terence—

> "Curemus æquam uterque partem; tu alterum,
> Ego item alterum."

One more quotation, and we are done.

"As such, then, my dear sir, I have ventured thus, while life is so short and art so long, to ask for myself a niche in the great edifice of surgical facts and truths of which you are the constructor and owner. I have to ask of you the privilege that I may be allowed within this storehouse, containing so much of the treasures of our science, to make safe deposit and registration of the detached fragments of the scaffolding at least, of that extended work of my own on Operative Surgery, which, as you are aware, I have for so long a time meditated, and of which these offerings must for the present be received as the pledge."

This is a gem in its way, for the equal of which we must refer to a quotation already made. Observe: M. Mott wants first a "niche" in Velpeau's "edifice"—a place to put his body, or his statue, or his "memory" after his kinsman's "balm" shall have descended upon it like dew from heaven upon a flower. But this will not do; so he likens his friend's beautiful edifice to a "store-house," and asks to throw into it the "fragments" (viz: loose planks and pieces of scantling) "of the scaffolding" of his own abandoned building. We repeat, the beauty and classic grace of this can be equalled by nobody but Townsend.

There are many other things in these prefaces deserving of notice, but we must forbear. The critical reader will see that, even in the sentences we have quoted, we have omitted many things worthy of remark. We need scarcely add that those who succeed so well in putting their own thoughts into English, succeed quite as well in translating the thoughts of another man from a foreign tongue into English. And now we wish to know if the men who thus write deliberately, and not in the hurry of supplying a periodical press, have not a right to "pride themselves on their work?" to congratulate themselves on their knowledge of the vernacular? to speak of "inexcusable and gross blunders" in other works? and to assume a high tone about the "depreciation of the standard of American education and character, by the

*book-making* attempts of certain adventurers in the profession?"

It was our intention now to take up the text, in order to fulfil the promise we made in the beginning; but the relentless tyrants of the printing-office tell us we must stop. We shall take the first opportunity to redeem this promise. For fear this may not be before the issue of the third volume, let us beg the translators, for the sake of good taste, and of the American press, the character of which has been so discredited, to make their translation less literal; to give English idioms in the place of French; to drop the French prefix *M.* before American and English names; not to place French words in brackets after giving the proper translation; and above all not to use a French word when there is an equivalent in English. If, in addition to this, they will remove the citations to the bottom of the page, and will never include in brackets more than one line between a verb and its nominative, an adjective and its noun, a preposition and the words to which it relates, they will improve the appearance and enhance the usefulness of these volumes.

Meantime, until we can spread something of it before our readers, we beg leave to assure them that despite the inconsiderate and silly laudations of the translator and editor, despite the bad taste which seems to have presided over the enterprise from the beginning, despite the want of grammatical knowledge everywhere displayed, the book is a good book, and a valuable book. It could have been written by no common man. It could not have been written at all in this country, simply for want of books from which to compile it. Yet it is by no means going to supercede every other book. As a text book, for which purpose it is so much vaunted, we should never think of placing it in the hands of a student. But more of this anon.

